# Physician marriage survey reveals sex and specialty differences in marital satisfaction factors

**DOI:** 10.1038/s41598-024-55437-3

**Published:** 2024-03-02

**Authors:** Rajeev R. Dutta, Anthony T. Wu, Bryce Picton, Saloni Shah, Michelle Chernyak, Kelly Bauer, Sean Solomon, Josephine Chang, Britney Nguyen, Mio Jiang, Anju Hurria

**Affiliations:** School of Medicine, University of California, Irvine, 101 The City Drive S, Orange, CA 92868 USA

**Keywords:** Physician marriage, Physician wellness, Children, Survey, Health care, Health occupations

## Abstract

Physician marriage is a valuable indicator of how vocational factors (e.g. work hours, stressors) impact satisfaction in relationships and physician wellness overall. Previous studies suggest that gender and specialty influence marriage satisfaction for physicians, though these often come from limited, local, cohorts. A cross-sectional survey was designed and distributed to publicly available email addresses representing academic and private practice physician organizations across the United States, receiving 321 responses (253 complete). Responses included data on demographics, medical specialty, age at marriage, stage of training at marriage, number of children, and factors leading to marital satisfaction/distress. A multivariable ordinal logistic regression was conducted to find associations between survey variables and marriage satisfaction. Survey results indicated that 86.5% of physicians have been married (average age at first marriage was 27.8 years old), and the rate of first marriages ending is at least 14.7%. Men had significantly more children than women. Physicians married at least once averaged 1.98 children. “Other” specialty physicians had significantly more children on average than psychiatrists. Marrying before medical school predicted practicing in private practice settings. Job stress, work hours, children, and sex were most frequently sources of marital distress, while strong communication, finances, and children were most frequently sources of marital stability. Sex differences were also found in distressing and stabilizing marital factors: Female physicians were more likely to cite their spouse’s work hours and job stress as sources of marital distress. Finally, surgery specialty and Judaism were associated with higher marriage satisfaction, whereas possession of an M.D. degree was associated with lower marriage satisfaction. This study elucidated new perspectives on physician marriage and families based on specialty, practice setting, and stage of training at marriage. Future studies may focus on factors mediating specialty and sex’s impact on having children and marriage satisfaction. To our knowledge, this study is the first physician marriage survey which integrates multiple factors in the analysis of physician marriages.

## Introduction

Physician marriage is of increasing importance as interest in physician wellness grows since marriages (and subsequent divorces) are sources both of stability and distress for physicians. One study suggests that the likelihood of a physician ever being divorced is 24.3% (with female physicians experiencing significantly higher odds of divorcing than male physicians)^1^.

Marriage satisfaction is a key factor in physician wellness since unmarried status has been associated with worse psychological symptoms for physicians, especially during infectious disease breakouts like the recent COVID-19 pandemic^2,3^. Further, marriage may be a protective factor against physician burnout^4^. Estimates on marriage satisfaction vary: one study suggests 63% of male physicians report high marital satisfaction while only 45% of female physicians report high marital satisfaction (though this difference may be moderated by age-adjustment) while another study estimates a satisfaction rate of 85% across genders and another (for dual physician marriage) estimates 86.8%^1,5–7^.

Marriage may not provide unilaterally positive effects on physician wellness, and these effects may be mediated by gender. Studies show that women physician-researchers spend significantly more time on household tasks compared to their male counterparts, which may contribute to role conflict and higher reported dissatisfaction with work-life balance for women physicians^8–10^.

Interpersonal dynamics depending on physician specialty may play a role in marriage and divorce. A study from data collected on a subset of The Johns Hopkins School of Medicine alumni suggested that physicians may be more or less likely to divorce depending on their specialty, estimating that psychiatrists had a 50% likelihood of being divorced, surgeons had a 33% likelihood of being divorced, and specialties like internal medicine, pediatrics, and pathology has a divorce rate of around 22–24%^11^. Why exactly divorce rate may vary between specialties is only speculative, but work hours and lifestyle may contribute to this difference in rate^11,12^. Still, some estimates of physician specialty work hours suggest that psychiatrists work among the fewest hours of all physicians, so it is somewhat unclear as to why a staggeringly higher rate of divorce was found^13^.

An additional area of interest is physician marriage benefits dependent on whether the spouse is also a physician. Women in physician-physician marriages have been found to work five fewer hours a week compared to other women physicians, and men in physician-physician marriages have found to work three fewer hours a week compared to other men physicians, which may indirectly contribute to the psychological benefits of physician marriage^14^.

However, these impacts may again not be exclusively beneficial. One study found that surgeons married to (or in a domestic partnership with) other physicians were more likely to delay having children, more likely to believe that having children had slowed their career advancement, and less likely to believe that they had enough time for their families compared to surgeons married to non-physicians^15^. These results were found to be even more extreme for surgeons married to other surgeons.

Furthermore, the decision to have children can impact physician wellness, possibly in a manner dependent on gender. Marriage and children for female physicians predict lower earnings and time spent practicing medicine compared to male physicians, possibly due to fewer hours worked on account of domestic responsibilities^16^. A survey of 5,582 physicians found that almost half of all physicians believed their career negatively impacted their relationship with their children. Both male and female physicians were significantly more likely to report experiencing a negative influence of their career on child-rearing compared to the American working population^17^.

The present survey-based study aims to contextualize findings across the literature using a survey to suggest new or evaluate previous characterizations regarding physician sex, specialty, age at marriage, stage of training at marriage, children from marriage, and more on marriage longevity and satisfaction.

## Methods

All data were collected anonymously, and this study was considered exempt by the University of California, Irvine’s Institutional Review Board (UCI IRB: #1931). All methods were performed in compliance with University of California, Irvine’s Human Research Protections guidelines. Informed consent statements were included at the beginning of the survey, which participants were required to read and agree to before taking the survey.

### Survey distribution

A cross-sectional survey was designed using Qualtrics XM software [Version February, 2023: https://www.qualtrics.com] and distributed to 2298 email addresses from publicly available internet sources representing academic and private medical centers (e.g. university hospital faculty listings, private clinic care team webpages) from across the United States. For example, a list of medical schools by state was procured, and relevant contact points for each medical school (e.g. research coordinator, public relations chairperson, communications) were added to the email list. For smaller, private physician groups, administrative or executive staff were often contacted. As a result, response rate and bias was likely complicated depending on dissemination (or lack thereof) within a given organization.

### Survey design

The survey collected data about each physician’s medical degree (e.g. M.D., D.O., M.B.B.S.), additional degrees, level of practice, practice setting (i.e. academic or private), medical specialty, marital status, number of marriages/divorces, stage of training (e.g. residency, attending, retired) along with demographic information including physician’s state of practice, race, religion, and year of birth.

For physicians that have been married, marriage year, stage of training at marriage, specialty of spouse (if physician), number of children, satisfaction, sources of stress, sources of stability, divorce or separation status, and year of divorce or separation (if applicable) for each marriage were collected. From this information, age at marriage and length of marriage were calculated, where applicable.

These questions were included in the survey for a variety of reasons, often in response to the previous (albeit limited) literature on physician marriage and satisfaction. For example, specialty choice was included since it was a central focus of the seminal Johns Hopkins study on physician divorce^11^. The emphasis on gender, children, and factors leading to stability or dissatisfaction in marriage were similarly motivated by previous studies^5,6,9,16^. Other variables, like practice setting and stage of training at marriage, were included because they were not well-represented in previous literature on physician marriage.

Thus, the survey was designed to contribute to a fuller understanding of factors previously investigated in physician marriage and satisfaction (by attempting to replicate earlier findings) while also providing new perspectives on some variables underexplored thus far (e.g. practice setting and stage of training at marriage).

### Responses and exclusion criteria

The survey was active through January and February 2023, during which 321 responses were collected (253 complete and included), resulting in a 14.0% response rate. This response rate may be inaccurate insofar as recipients may have forwarded the survey within their institutions and some recipient email addresses may have no longer been actively monitored at the time of survey distribution. Responses were excluded if the respondent did not report possessing a medical degree or did not complete all required questions, including questions about whether the respondent is married and the respondent’s current level of practice.

### Data analysis

Using the SciPy 1.12.0 [https://scipy.org], SciKit Learn 1.4.1 [https://scikit-learn.org/stable/], StatsModels 0.14.1 [https://www.statsmodels.org/stable/index.html], Mord 0.7 [https://pypi.org/project/mord/], and ResearchPy 0.3.5 [https://pypi.org/project/researchpy/] libraries in Python 3, chi-square analysis or Analysis of Variance (ANOVA) was performed (by R.D. and A.W.) as appropriate to analyze data from survey responses (α = 0.05). In addition to survey values, the age of each respondent at their first marriage and total number of children were calculated. Additionally, specialties were aggregated into the following categories after collection using National Resident Matching Program specialties (plus urology and ophthalmology): family medicine, internal medicine, pediatrics, psychiatry, surgery (including obstetrics/gynecology and urology) and “other” specialties (including neurology, pathology, anesthesiology, emergency medicine, dermatology, etc.). These aggregated categories were established to avoid attaining false statistical significance from low-powered subgroup analyses.

The chi-square and ANOVA tests were applied using independent variables like age, specialty, current level of practice, and gender compared to dependent variables like number of children and satisfaction in marriage ("Yes” or “No”). Factors like practice setting (i.e. academic or private) served as independent or dependent variables according to which association was under investigation (e.g. practice setting could be an independent variable for satisfaction in marriage and a dependent variable with respect to specialty). If chi-squared analysis on nominal variables was significant, pairwise chi-squared analysis with Holm-Sidak correction was done to determine which categories had significant differences.

### Multivariable analysis

We conducted a multivariable regression, considering survey items that received over 150 responses. Only responses encompassing all considered variables were retained for further examination. To facilitate analysis, variable aggregation was performed (e.g. adding the number of children across marriages to arrive upon the total number of children per physician). Additionally, variables for which all responses were the same were filtered out, since these variables would not, by themselves, contribute to the resulting analysis. For nominal variables, we adopted a one-hot encoding strategy. To address the issue of perfect separation resulting in singular matrices due to one-hot encoding, we excluded the category with the fewest participants for each nominal variable from the analysis. Binary variables were represented as [0, 1], while ordinal variables were encoded as integers maintaining temporal coherence (e.g. for the current level of practice, "resident" preceded "fellow"). Marriage satisfaction was used as the dependent variable and was also encoded ordinally, reflecting increasing satisfaction levels (i.e. not satisfied, somewhat satisfied, satisfied). Ratio data, being inherently numerical, underwent no additional encoding.

To mitigate high collinearity, we calculated the variance inflation factor (VIF) for each candidate feature. Features with the highest VIF were iteratively dropped from the analysis to account for systemic multicollinearity changes if its VIF exceeded the standard threshold of 5.

For modeling, we used an ordinal logistic regression (All-threshold variant) to determine feature coefficients. To enhance model robustness and prevent overfitting, we applied ridge regularization with α = 0.01. Coefficient significance was assessed using the Wald test (α = 0.05).

## Results

### Demographic information

See Table 1 for full demographic data. Of the completed and included survey submissions, 57.4% of respondents (144) identified as female. Respondents spanned 34 United States/territories and were 76.3% white (193), with Asian (9.5%, 24) and Black (6.3%, 16) composed the next most represented races. Hispanic or Latino physicians made up 8.8% of respondents.

**Table 1:** Demographic and practice data for respondents.

Category (# of respondents)			n	%
Demographics	Sex (251)	Female	144	57.4%
		Male	105	41.8%
		Prefer not to say	2	0.8%
	Race (253)	White or Caucasian	193	76.3%
		Asian	24	9.5%
		Black	16	6.3%
		American Indian/Alaska Native	1	0.4%
		Other	10	4.0%
		Two or more races	6	2.4%
		Prefer not to say	3	1.2%
	Ethnicity (250)	Hispanic or Latino	22	8.8%
		Not Hispanic or Latino	228	91.2%
Education	Medical degree (253)	M.D	212	83.8%
		D.O	39	15.4%
		M.B.B.S	2	0.8%
	Additional degrees (56)	M.B.A	8	14.3%
		M.P.H	13	23.2%
		M.S	22	39.3%
		Ph.D	5	8.9%
		Other	11	19.6%
Practice	Level of practice (251)	Resident	38	15.1%
		Fellow	4	1.6%
		Attending	193	76.9%
		Retired	16	6.4%
	Practice setting (252)	Academic	163	64.7%
		Private practice	89	35.3%
	Specialty (249)	Family medicine	57	22.9%
		Internal medicine	33	13.3%
		Pediatrics	27	10.8%
		Psychiatry	37	14.9%
		Other specialty	51	20.5%
		Surgery	44	17.7%

Number of respondents for survey questions and distribution of responses.

### Education

All participants possessed a medical degree: 212 (83.8%) had a Doctor of Medicine degree (M.D.), 39 (15.4%) had a Doctor of Osteopathic degree (D.O.), and 2 (0.8%) had a Bachelor of Medicine, Bachelor of Surgery degree (M.B.B.S.). Less than a quarter (22.2%) of respondents had an additional degree, like a Doctor of Philosophy (Ph.D.) or Master’s degree.

### Practice information

The respondents occupied a spectrum of the medical training pathway: 38 (15.1%) were resident physicians, 4 (1.6%) were fellows, 193 (76.9%) were attending physicians, and 16 (6.4%) were retired. Just under two-thirds (64.7%) of respondents practiced in an academic setting, while the remaining third (35.3%) were in a private practice setting.

After specialty aggregation, it was found that 57 respondents were in family medicine (22.9%), 33 were in internal medicine (13.3%), 27 were in pediatrics (10.8%), 37 were in psychiatry (14.9%), 51 were in the “other” specialty category (20.5%), and 44 were in surgery (17.7%).

### Marriage and relationships

**Figure 1:**
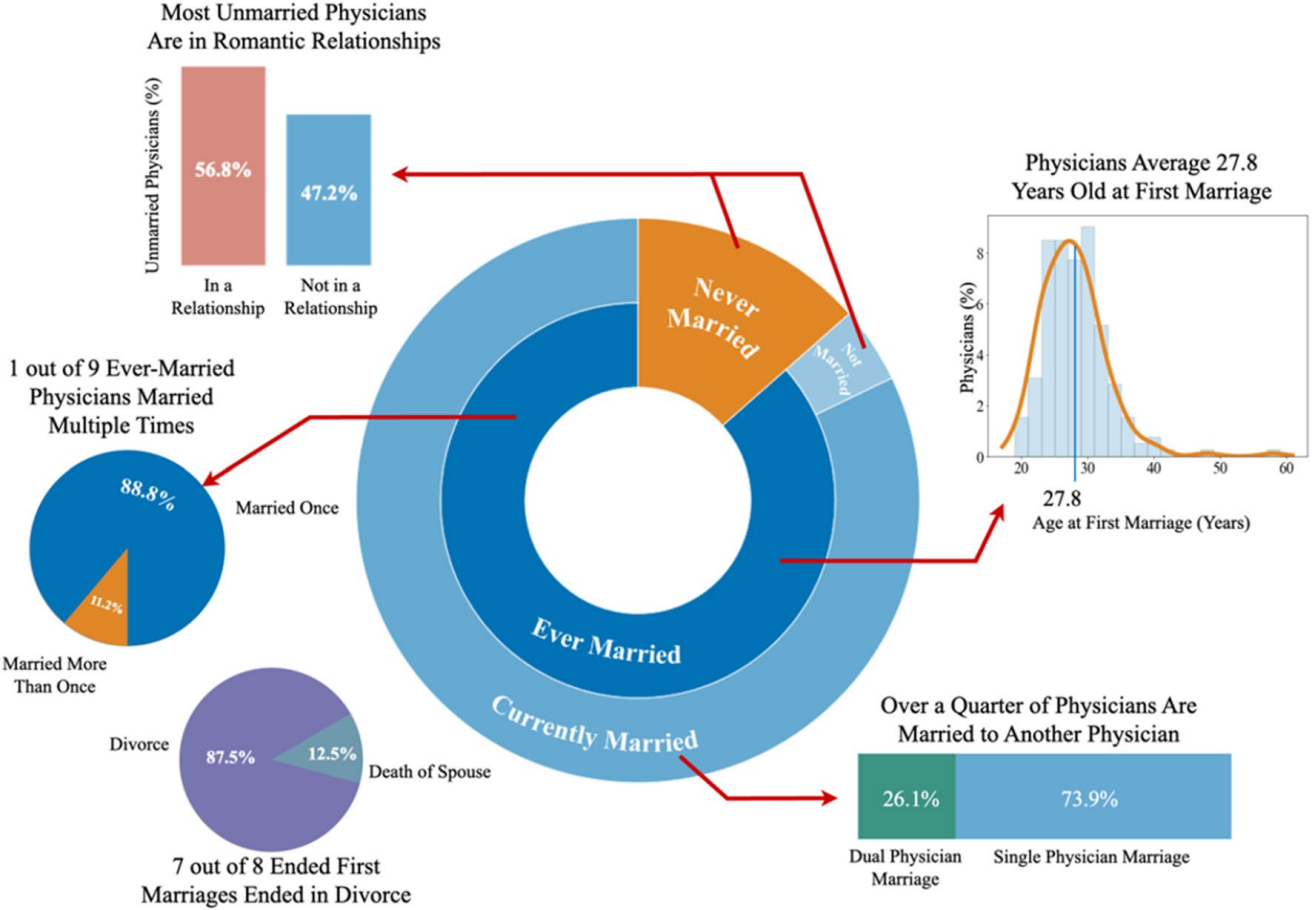
Overview of respondents’ marital characteristics. Visualization of distribution of currently married, ever married, never married, and not currently married respondents. Physicians are 27.8 years old on average at their first marriage and 26.1% are currently married to another physician. One out of nine physicians ever-married physicians have been married multiple times, and seven out of eight ended first marriages were a result of divorce, rather than death of the spouse. Over half of unmarried physicians are in romantic relationships.

Of 252 respondents to questions about marriage, 218 (86.5%) were either currently married or previously married. The average age of a physician at their first marriage was 27.8 years old. Currently married physicians composed 207 (82.1%) of total respondents. One quarter of currently married physicians were married to another physician (26.1%). Dual physician marriages were not significantly more or less likely to end in divorce than other physician marriages. Over half (56.8%) of currently unmarried physicians were in a romantic relationship. Survey results showed that 14.7% of ever-married physicians have at least once been in a marriage that has ended, and 11.2% have been married more than once. Of first marriages that ended, 87.5% (28 of 32) ended in divorce and the remaining 12.5% (4 of 32) ended in the death of the spouse. See Fig. 1 for a visual overview of the marital characteristics of respondents.

### Children

**Figure 2:**
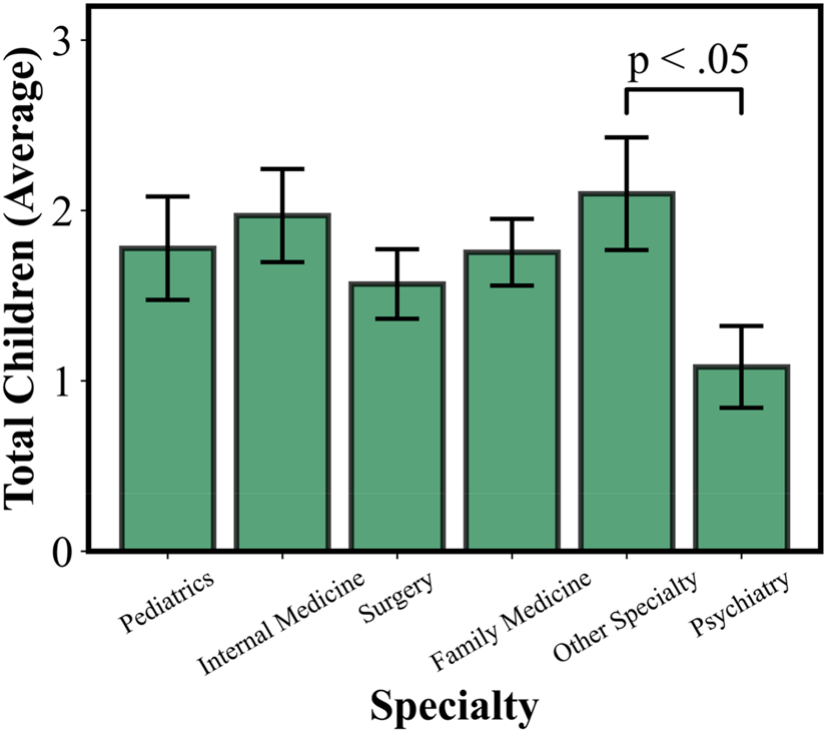
Specialty and average total number of children physicians had across all marriages. “Other” specialty physicians were found to have more children, on average, than psychiatry specialization physicians.

See Fig. 2 for a summary of specialty choice’s association with the number of children physicians have. On average, physicians married at least once had around two children (1.98) overall. In addition to the aforementioned associations involving children (private practice physicians were more likely to have children and had more children on average than academic physicians, “other” specialty physicians were more likely to have children and had more children on average than psychiatrists), several more associations involving children were found.

For example, physicians who identified themselves as male had more children, on average, than physicians who identified themselves as female, both in total (*p* < 0.001, F = 12.2) and in the current marriage (*p* = 0.006, F = 7.6). The stage of training in which a physician married influenced the number of children they had from that marriage (*p* = 0.002, F = 5.0). Physicians married before medical school had the most children (*μ* = 2.79, n = 34), physicians married during residency had the next most children (*μ* = 1.93, n = 54), then physicians married during medical school (*μ* = 1.64, n = 61), and finally physicians married as an attending (*μ* = 1.61, n = 41). This pattern closely follows, but does not match, ascending average age per stage of training (which, youngest to oldest, was before medical school, during medical school, during residency, and as an attending).

### Specialty

No significance was found in specialty choice’s influence on marriage satisfaction, number of children, or likelihood of divorce. While the family medicine and psychiatry specialties each reported the highest number of divorces (6 each, 10.5% of family medicine physicians and 16.2% of psychiatrists), no statistically significant correlations between specialty choice and marriage outcome were found. Similarly, no significant differences between specialty choice and whether a physician is satisfied with their marriage was found. Specialty choice did not appear to correlate with the stage of training at which a physician got married.

However, physicians in the “other” specialty (e.g. neurology, anesthesia, dermatology) category had a significantly higher average number of children than psychiatrists across all marriages (*p* = 0.02, F = 5.4) and current marriage (*p* = 0.04, F = 4.4). These “other" specialty physicians were also significantly more likely to have children from their current marriage than psychiatrists (*p* = 0.02, χ^*2*^ = 11.5) No such associations were found between any other pair of specialties.

### Practice setting

**Figure 3:**
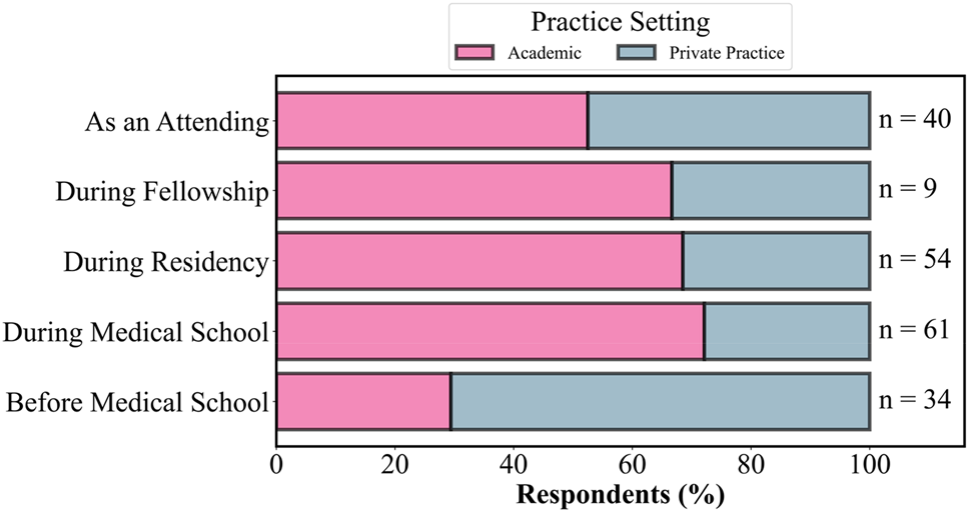
Practice setting with respect to stage of training at marriage. Physicians married before medical school were significantly more likely to practice in a private practice setting compared to physicians married during medical school or residency, who were more likely to practice in an academic setting (*p* = *.*03, χ^2^ = 19.7).

Practice setting (private versus academic) was not associated with more marriages or divorces. However, practice setting was found to be associated with the average number of children physicians have, with private practice physicians having significantly more children on average than academic physicians (*p* < 0.001, F = 46.6) and more likely to have children from their current marriage (*p* = 0.02, χ^2^ = 11.4) than academic physicians*.* The stage of training in which a physician married (e.g. before medical school, during residency, etc.) varied significantly with the practice setting (private or academic) of the physician (*p* = *0.0*3, χ^2^ = 19.7). Physicians married during residency were significantly more likely to practice in an academic setting than physicians married before medical school (*p* = 0.01, χ^2^ = 12.8). See Fig. 3.

### Satisfaction

Of 199 respondents, 174 (87.4%) physicians reported being satisfied with their marriages, 3 (1.5%) physicians reported being dissatisfied with their marriage, and 22 (11.1%) physicians reported being somewhat satisfied with their marriages.

**Figure 4:**
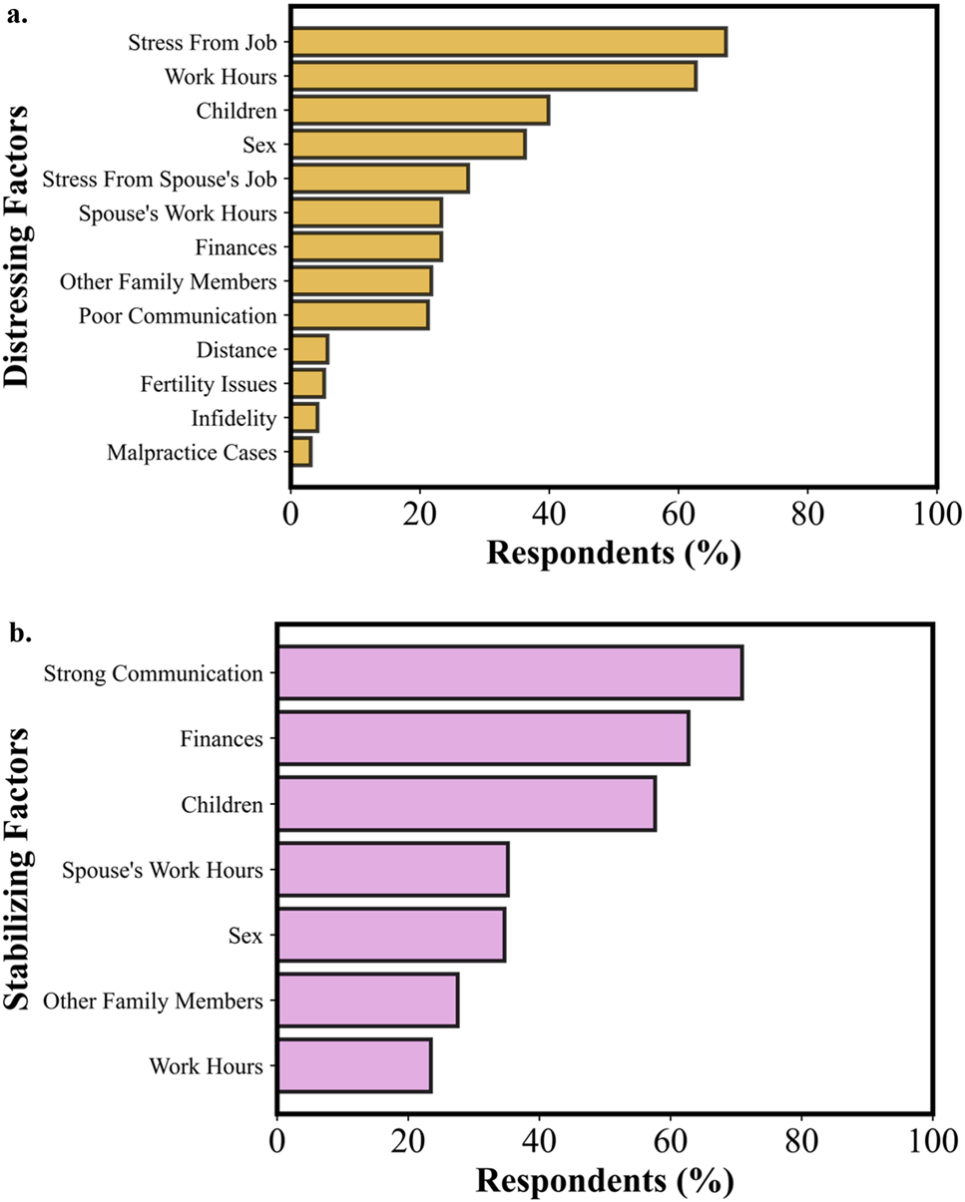
Factors contributing to marital distress (**a**) and marital stabilization (**b**). In descending percentage of respondents, stress from the physician’s job, the physician’s work hours, children, and sex were the most selected distressing factors for marriage. Strong communication, finances, children, and the spouse’s work hours were the most selected stabilizing factors for marriage. Respondents could select multiple options for each question.

The most common sources of stress that physicians named for their marriage were stress from their job (67.4%), their work hours (62.7%), children (39.9%), and sex (36.27%). The most common sources of marriage stability were strong communication (70.9%), finances (62.8%), and children (57.7%). See Fig. 4 for a summary of the distressing and stabilizing factors recorded in the survey.

**Figure 5:**
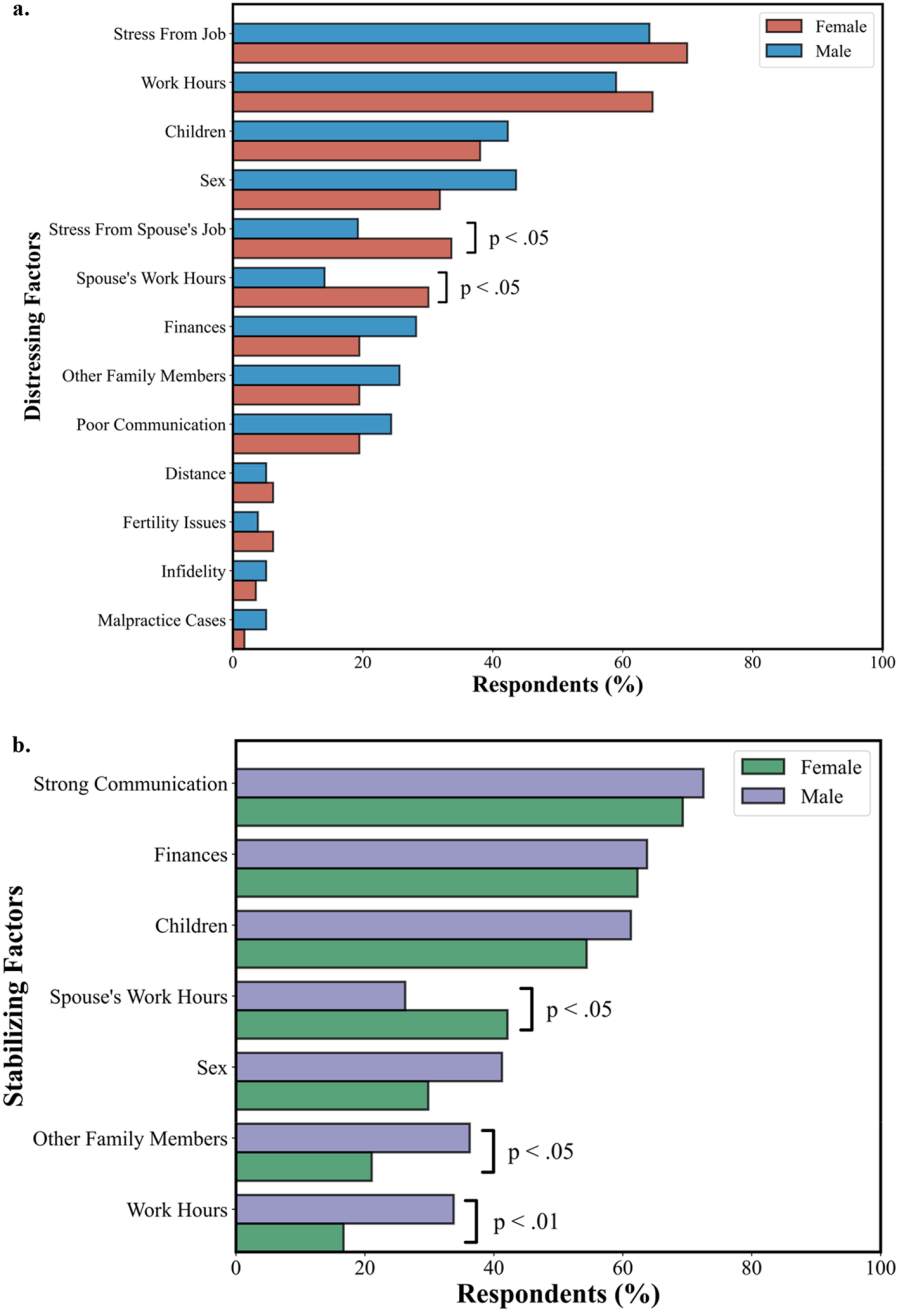
Percent of female and male physicians selecting factors contributing to marital distress (**a**) and marital stability (**b**). Female physicians were significantly more likely than male physicians to cite their spouse’s job and work hours as sources of distress. Male physicians were significantly more likely than female physicians to select work hours and other family members as sources of stability and significantly less likely to select their spouse’s work hours as a source of stability.

**Figure 6:**
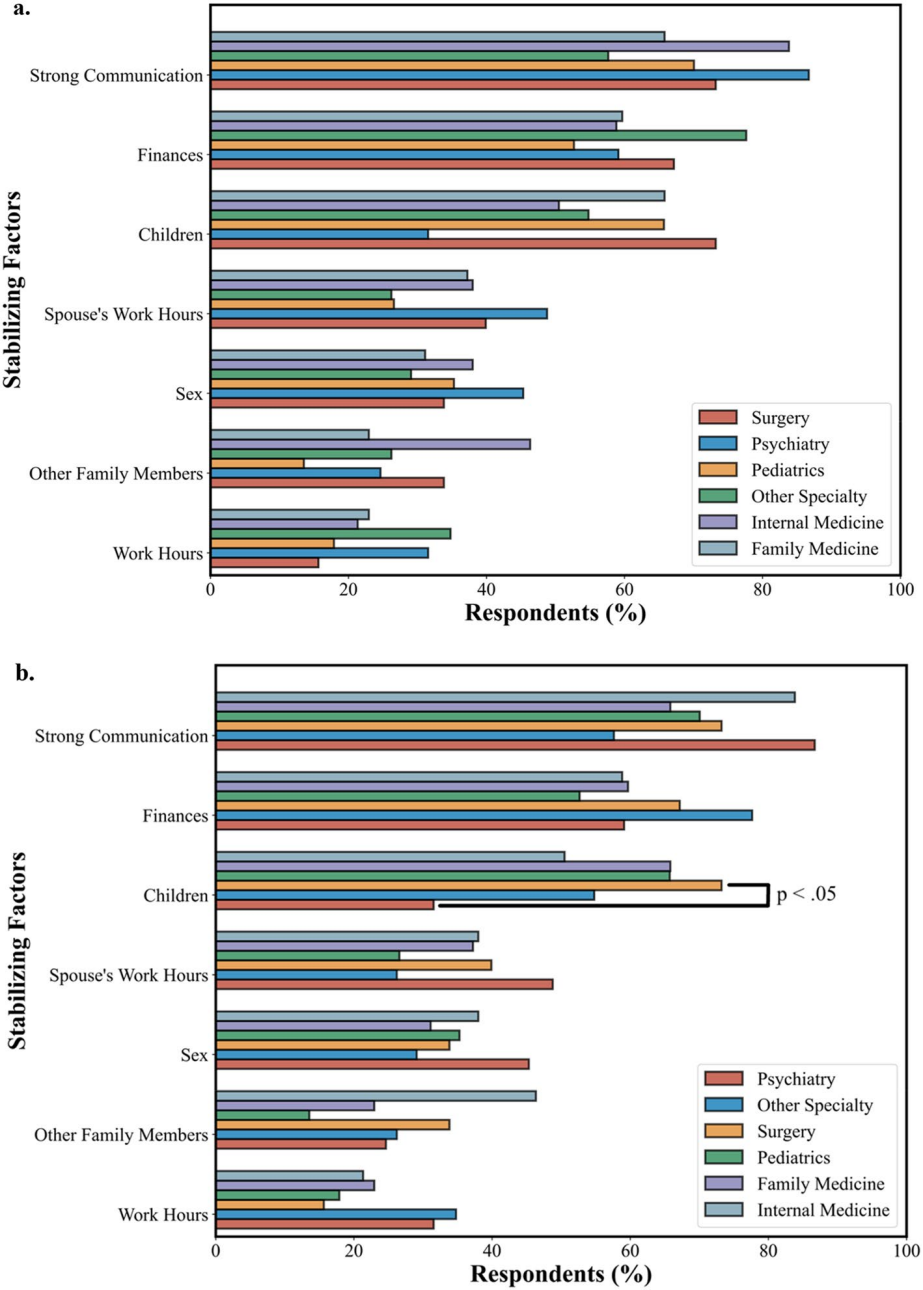
Factors contributing to marital distress (**a**) and marital stability (**b**) based on percentage of respondents in each specialty. Surgeons were significantly more likely than psychiatrists to select children as a source of stability.

Distressing and stabilizing factors were also analyzed as a function of sex and specialty (see Figs. 5 and 6). Female physicians were significantly more likely than male physicians to select their spouse’s work hours (*p* = 0.02, χ^2^ = 5.7) and stress from their spouse’s job (*p* = 0.04, χ^2^ = 4.1) as sources of marital distress. Conversely, male physicians were significantly more likely to select other family members (*p* = 0.03, χ^2^ = 4.7) and their work hours (*p* = 0.01, χ^2^ = 6.7) as sources of marital stability. However, female physicians were significantly more likely to select their spouse’s work hours as a source of marital stability (*p* = 0.03, χ^2^ = 4.5).

Significant differences in findings for stabilizing and distressing factors between specialties were not found, with one exception. Surgeons were significantly more likely than psychiatrists to select children as a source of marital stability (*p* = 0.02, χ^2^ = 9.2).

### Multivariable analysis

**Figure 7:**
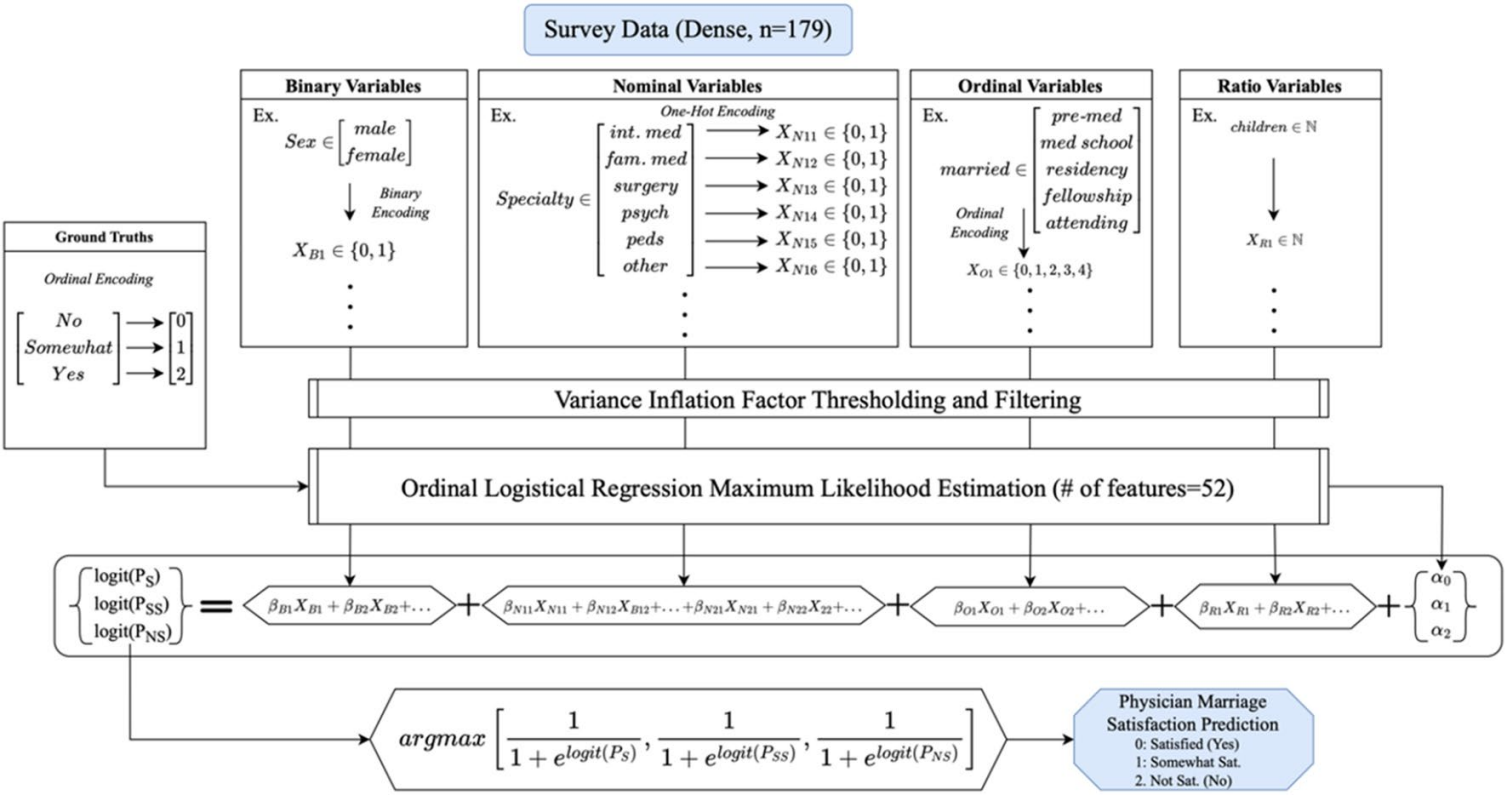
Results of the ordinal logistic regression. Binary, nominal, and ordinal variables were coded to generate coefficients with a dependent variable of physician marriage satisfaction.

See Fig. 7 for a visualization of the ordinal logistic regression. We identified 15 dependent variables for inclusion in our multivariable analysis. From the dataset, 179 responses addressed all dependent variables and were consequently incorporated into the analysis. Subsequent to variable encoding and exclusion based on variance inflation factor thresholding, a total of 52 features were retained for the ordinal logistic regression.

Among these features, four exhibited statistical significance: possessing an M.D. degree (coefficient = − 5.64, *p* < 0.00001), specializing in surgery (coefficient = 3.89, *p* = 0.01), and affiliating with Judaism (coefficient = 4.69, *p* = 0.01) or an unreported/other religion (coefficient = 4.21, *p* = 0.03). Positive coefficients indicate positive contributors to marriage satisfaction, while negative coefficients indicate negative contributions to marriage satisfaction. For example, the results suggest that specializing in surgery is positively associated with high marriage satisfaction, whereas possessing an M.D. degree (i.e. rather than a D.O. or M.B.B.S. degree) is negatively associated with high marriage satisfaction (i.e. M.D. degree is associated with lower marriage satisfaction).

## Discussion

The wide range of findings from this survey helps contextualize other findings in the literature while adding new perspectives to satisfaction, specialty, sex, practice setting, and stage of training at marriage, and children on one another.

The rate of a physician marriage ending found in this study (11.2%) is lower than the rate of divorce of the general population and other professions, which some studies have corroborated^18^. Factors leading to this increased stability may include older age at marriage and higher socioeconomic status^19^.

### Children

The effect of sex on having children was also impactful for understanding physician wellness. The finding that male physicians had significantly more children on average than female physicians and were significantly more likely to have children than their female counterparts may highlight a disproportionate burden on women in child-rearing than on men. Role conflict (mediated by work hours) has been suggested to mediate marital and parental satisfaction in physicians, so the additional difficulty for women physicians to have children is an important opportunity to evaluate the structural factors (e.g. paid time off, potential career obstacles from taking sufficient time off, childcare availability) hindering a critical facet of physician wellness for women^5^. In concert with previously explored associations between having children and factors involved in physician marriage satisfaction (e.g. fewer work hours), our findings suggest that male physicians, physicians in private practice, and physicians in “other” specialties may disproportionately benefit from having children as contributors to marital stability^14,16^. By contrast, other factors involved with child-rearing, including lower earnings and strain from conflict between professional and personal responsibilities, may also impact the identified groups, though these factors for distress often fall on female physicians^15,17^.

### Specialty

Strikingly, the specialty-focused findings of this study contrast with findings in a study of Johns Hopkins graduates which suggested that the rate of divorce for psychiatrists was 50% (our study estimates 16.2%) and 33% for surgeons (our study estimates 4.5%)^11^.

Additionally, no statistically significant differences between specialties and the rate of divorce were found in the present study. Deviations from the earlier study may be a consequence of confounding factors (e.g. social change during the time of study participant’s marriages, restricted location of study, cohort similarities). Sample size differences between the studies may also contribute to deviations in their findings.

However, specialty choice was found to vary with other factors related to marriage. This study found that physicians in “other” specialty fields were significantly more likely to have children and had significantly more children on average than psychiatrists. Factors like differences in pay, work hours, and sex distribution between the specialties may mediate this association. Most of the psychiatrists in this study (91.9%) practice in an academic setting (rather than private practice) which, although not representative of the United States population of psychiatrists, may explain the fewer number of children reported in this study. This finding from the study suggests which groups of physicians (i.e. those in “other” specialties) are likely to experience the benefits and stressors associated with having children that have been identified in previous studies^14–17^.

### Satisfaction

The high rate of marital satisfaction (87.4%) found in this study matches similar studies, which have estimated physician marital satisfaction rates of 85% and 86.8% (the latter for dual physician marriages), which contradict an archetypal view of dysfunctional physician marriage, supported by the findings of previous studies^6,7^. The factors influencing marital distress being primarily stress from work and work hours echoes findings of studies which suggests that quality time spent with the spouse increase marital satisfaction^19^. Children and sex were the next most common sources of distress, which emphasizes the challenges of role conflict and intimacy in marital stability, especially for women^7,8^. Sources of marital stability predominantly consisted in strong communication, financial stability, and children, which also corroborates other studies on marriage satisfaction^7,20–22^.

The sex analysis of factors in marital distress and stability was particularly interesting in that female physicians were significantly more likely than male physicians to list their spouse’s work hours as both a source of distress and as a source of stability. The finding could suggest that the impact of the works hours of a male physician’s spouse tend not to particularly valence the physician’s perception of marital stability and distress. This tendency may echo traditional gender roles with respect to child rearing and paid labor^9^. The finding that male physicians were significantly more likely than female physicians to list other family members as a source of marital stability may suggest that male physicians perceive a relatively high contribution to childcare or other familial support systems.

These findings, overall, precisify previous observations about the relatively high rate of physician marital satisfaction, suggesting the features of physician marriages that contribute to marriage stability, including those mediated by sex.

### Multivariable analysis

The significant findings of the ordinal logistic regression suggested interesting contributors to physician marriage satisfaction. That the possession of an M.D. degree is negatively associated with satisfaction is puzzling—since the factor is not obviously or significantly mediated by another factor (e.g. specialty choice, stage of training at marriage, etc.), interpretations of the finding are speculative. However, the finding that surgery specialty is positively associated with marriage satisfaction has been suggested by previous studies, though it also contrasts with other studies that suggest that surgeons delay family life for their careers^11,15^. Finally, the finding that physicians following Judaism or another, unspecified religion were positively associated with marriage satisfaction was striking. The role of religion and religious communities in marital satisfaction remains a ripe area for investigation.

### Limitations and future directions

Limitations of the present study include a skew toward academic physicians rather than private practice physicians in responses, which could be a result of response bias for academic practitioners, as well as lower visibility of private practice email addresses online. A relatively smaller sample size and response rate of this study is also a limitation, potentially impairing the reliability of smaller analyses in this study. The geographic and cross-sectional age diversity is a strength of the study, mitigating bias from limited location and age backgrounds. However, despite representing 34 of the United States, the small sample size limits representativeness and the generalizability of findings. Further, the method of email address acquisition and relatively short window for survey collection poses a challenge to replicability of the study.

Future studies on physician marriage and wellness might explore specific reasons for the gradient in men and women’s ability to have children as physicians, as well as changes that might ameliorate this difficulty. The stage of training at marriage’s impact on eventual practice setting is also of further interest. Investigating what factors (e.g. work hours, practice autonomy, etc.) of private practice favors physicians married earlier in training could be of considerable interest in understanding physician wellness dependent on practice setting.

Physician marriage and its impact on wellness is a relatively young field of study that merits careful study to elucidate multiple factors impacting career and life satisfaction and happiness for physicians. This study was the first nationwide survey to our knowledge that considered a wide variety of factors, including age, gender, stage of training at marriage, practice setting, current stage of training, specialty, number of children, and more, on marriage satisfaction and longevity.

## Conclusion

This survey sought to characterize properties of physician marriages, including specialty choice, children, and factors leading to marital distress and stability. The study identified that specialty choice, male sex, and private practice setting is associated with having more children on average, which was often identified as a source of stability in physician marriages. Further, job stress and work hours were most frequently identified as sources of distress in physician marriages, whereas strong communication and finances were most frequently identified as sources of stability in physician marriages. Jointly, these findings strengthen the understanding of married physicians, highlighting the attributes of physicians associated with distress and stability in familial life.

### Supplementary Information

Supplementary Information.

## Data Availability

All data generated or analyzed during this study are included in Supplementary Files.
